# The Assessment of Infection Risk in Patients with Vitiligo Undergoing Dialysis for End-Stage Renal Disease: A Retrospective Cohort Study

**DOI:** 10.3390/pathogens13010094

**Published:** 2024-01-21

**Authors:** Pearl Shah, Mitchell Hanson, Jennifer L. Waller, Sarah Tran, Stephanie L. Baer, Varsha Taskar, Wendy B. Bollag

**Affiliations:** 1 Department of Medicine, Medical College of Georgia at Augusta University, Augusta, GA 30912, USA; peshah@augusta.edu (P.S.); mihanson@augusta.edu (M.H.); sarahytran@gmail.com (S.T.); stephanie.baer@va.gov (S.L.B.); vtaskar@augusta.edu (V.T.); 2 Division of Biostatistics and Data Science, Department of Population Health Sciences, Medical College of Georgia at Augusta University, Augusta, GA 30912, USA; jwaller@augusta.edu; 3 Charlie Norwood VA Medical Center, Augusta, GA 30904, USA; 4 Department of Dermatology, Medical College of Georgia at Augusta University, Augusta, GA 30912, USA; 5 Department of Physiology, Medical College of Georgia at Augusta University, Augusta, GA 30912, USA

**Keywords:** vitiligo, ESRD, bacteremia, septicemia, conjunctivitis, cellulitis, herpes zoster

## Abstract

Vitiligo is an autoimmune condition that causes patchy skin depigmentation. Although the mechanism by which vitiligo induces immunocompromise is unclear, other related autoimmune diseases are known to predispose those affected to infection. Individuals with vitiligo exhibit epidermal barrier disruption, which could potentially increase their susceptibility to systemic infections; patients with renal disease also show a predisposition to infection. Nevertheless, there is little research addressing the risk of infection in dialysis patients with vitiligo in comparison to those without it. A retrospective analysis was performed on patients with end-stage renal disease (ESRD) in the United States Renal Data System who started dialysis between 2004 and 2019 to determine if ESRD patients with vitiligo are at an increased risk of bacteremia, cellulitis, conjunctivitis, herpes zoster, or septicemia. Multivariable logistic regression modeling indicated that female sex, black compared to white race, Hispanic ethnicity, hepatitis C infection, and tobacco use were associated with an enhanced risk of vitiligo, whereas increasing age and catheter, versus arteriovenous fistula, and access type were associated with a decreased risk. After controlling for demographics and clinical covariates, vitiligo was found to be significantly associated with an increased risk of bacteremia, cellulitis, and herpes zoster but not with conjunctivitis and septicemia.

## 1. Introduction

Vitiligo, an autoimmune skin condition, impacts nearly 3 million Americans, with approximately 40% of adult vitiligo cases remaining undiagnosed [1]. The pathogenesis of vitiligo relates to the destruction of melanocytes by innate and adaptive immunological pathways, with an involvement of oxidative stress and pro-inflammatory cytokines, disruption of melanocyte adhesion, and dysregulation of CD8+ T-cells, resulting in patchy dyspigmentation of affected lesion sites [2,3,4]. As a result, vitiligo can negatively impact patients’ quality of life, especially those with higher body surface area coverage [5], resulting in an increased risk of psychiatric disorders including major depressive disorder [6,7]. Vitiligo is also associated with other comorbid autoimmune conditions, like diabetes mellitus, and connective tissue diseases, like discoid lupus erythematous, as well as ocular and psychiatric conditions [8]. Studies have demonstrated that patients with vitiligo are at an increased risk of obesity and renal diseases, such as chronic kidney disease and end-stage renal disease (ESRD) [7,9].

Patients with ESRD require dialysis or kidney transplants as a treatment. Patients undergoing dialysis are at an increased risk for systemic infections due to vascular and catheter access, as well as native and acquired immunosuppression [10,11]. Sepsis and other infections are the second leading cause of death in these patients, following cardiovascular disease [12,13]. Patients with vitiligo also have a disruption of their epidermal barrier, potentially making them more susceptible to systemic infections through this compromised barrier [14,15,16,17].

Based on the characteristic pathogenesis of vitiligo, it is reasonable to hypothesize that vitiligo, as a comorbidity in patients with ESRD undergoing dialysis, may lead to an increased risk of infections compared to the general population, potentially due to epidermal barrier dysfunction. However, there is a lack of research examining the correlation between vitiligo and infection risk in patients on dialysis. To address this gap in knowledge, we queried the United States Renal Data System (USRDS) for patients undergoing dialysis with a diagnosis of vitiligo to analyze whether vitiligo serves as an independent risk factor for bacteremia, septicemia, cellulitis, herpes zoster, and conjunctivitis in these patients.

## 2. Materials and Methods

### 2.1. Dataset Study and Cohort

The USRDS, funded by the National Institute of Diabetes and Digestive and Kidney Diseases, is a national data system that collects, analyzes, and distributes information about chronic kidney disease (CKD) and ESRD in the United States (US). The USRDS collaborates with organizations including the Centers for Medicare & Medicaid Services (CMS), the United Network for Organ Sharing (UNOS), and the ESRD networks to produce a dataset that includes demographics and CMS medical claims submitted to Medicare for all US patients on dialysis; all US patients undergoing dialysis are automatically enrolled in Medicare. In this study, the USRDS database was used to determine whether vitiligo is an independent risk factor for some infections in patients with ESRD. This research was deemed not Human Subjects Research by the Augusta University Institutional Review Board (reference #1592144-1).

Individuals in the USRDS from 18 to 100 years of age were eligible for inclusion in this study if they initiated dialysis between 2004 and 2019. Those who were less than 18 years or greater than 100 years of age, or had missing or unknown data on age, race, sex, ethnicity, access type, or dialysis type were excluded. Patients with ESRD with a diagnosis of vitiligo were identified in this database using International Classification of Disease (ICD)-9 (709.01) and ICD-10 (H02.73-H02.739 or L80) codes. The total sample size included was 1,526,270, and the analysis was performed to compare those with and those without a diagnosis of vitiligo.

### 2.2. Study Design

A retrospective cohort design was employed using data from the USRDS to analyze vitiligo and its association with different infectious outcomes in patients with ESRD.

### 2.3. Outcome Variables

Infectious outcomes of interest included bacteremia, cellulitis, conjunctivitis, herpes zoster, and septicemia [12,13,18,19,20]. Infections following the incident date of dialysis were determined using ICD-9 and ICD-10 codes from hospital, detailed, and physician/supplier claims. The person-years-at-risk was determined as the difference between the first date of the specific infection diagnosis and the incident date of dialysis. For those without an infectious outcome, this value was ascertained to be the difference between the first date of dialysis and either death or 31 December 2019.

### 2.4. Main Independent Variable—Vitiligo Diagnosis

A diagnosis of vitiligo after the incident date of dialysis was determined from hospital, detailed, and physician/supplier claims using ICD-9 and ICD-10 codes.

### 2.5. Demographic and Other Clinical Risk Factors

Demographic data including age, race, sex, ethnicity, dialysis modality, and access type were determined from the patient data file or CMS Form 2728. Per the instructions for the CMS 2728 form, both ethnicity and race are self-reported; the form itself is completed by the physician, likely in conjunction with the patient or the patient’s representative. Tobacco use (ICD-9: 305.1, V15.83l; ICD-10: Z72.0, F17.220), alcohol dependence (ICD-9: 303.90–303.93 and 305.00–305.03; ICD-10: F10. 2), and hepatitis C infection (ICD-9: 070.41, 070.44, 070.51, 070.54, 070.70, 070.71; ICD-10: B17.10, B17.11, B18.20, B19.20, B19.21) were ascertained from hospital, detailed, or physician suppler claims using ICD-9 and ICD-10 codes.

### 2.6. Statistical Analysis

Statistical analyses were performed using SAS 9.4 (SAS, Inc., Cary, NC), with statistical significance assessed using an alpha level of 0.05. Descriptive statistics including frequencies and percentages or means and standard deviations, where appropriate, for all variables were determined, overall, by vitiligo status and by each type of infection.

Logistic regression was used to examine the association of each demographic or clinical risk factor with vitiligo, and to examine the association of vitiligo, as well as each demographic and clinical risk factor, with each infection. An offset parameter of the natural log of the number of person-years-at-risk value was used in the estimation of the relative risk. For vitiligo or for each infection, each risk factor was assessed in a simple, bivariate model. All risk factors were then entered into a comprehensive full logistic regression model for the vitiligo outcome or for each infectious outcome, and a backward model building strategy was used to create the comprehensive final model, as previously described in Schwade et al. [21] and Momin et al. [22].

## 3. Results

Table 1 displays the overall descriptive statistics and the vitiligo status of patients, as well as the results of the simple and final logistic regression models on vitiligo, to assess potential correlates or confounders. Briefly, the average age of all 1,526,270 subjects with ESRD was 63.5 years (SD = 14.9), with the majority being white (66%) and male (57.2%). Nearly all (99.9%) subjects were on hemodialysis and 80.8% had a catheter for their access type. Only 676 subjects (0.04%) had a diagnosis of vitiligo. The dialysis modality was unable to be examined in logistic regression models as all subjects with vitiligo were on hemodialysis. All other demographic and clinical variables were associated with vitiligo in simple models, and the final multivariable logistic regression model indicated that black compared to white race, female sex, Hispanic ethnicity, tobacco use, and hepatitis C infection were associated with an increased risk of vitiligo, whereas increasing age and catheter access type compared to arteriovenous fistula (AVF) were associated with a decreased risk of vitiligo.

Table 2 provides the descriptive statistics of all variables by bacteremia, septicemia, cellulitis, herpes zoster, and conjunctivitis. The percentage with vitiligo was higher among those with each type of infection compared to those without the specific infection.

Table 3 presents the results of the simple models and the final model, examining the association of vitiligo with each type of infection when controlling for demographic and clinical covariates. In simple logistic regression models, vitiligo was associated with an increased risk of bacteremia, septicemia, cellulitis, and herpes zoster but not conjunctivitis. Controlling for demographic and clinical covariates, vitiligo remained associated with an increased risk of bacteremia, cellulitis, and herpes zoster but was no longer significantly associated with septicemia; vitiligo again showed no association with conjunctivitis. Increasing age, female sex, and catheter compared to AVF access, tobacco use, hepatitis C, and all other races compared to the white race were associated with an increased risk for all five infectious outcomes. Hemodialysis compared to peritoneal dialysis was associated with an increased risk of bacteremia, septicemia, and cellulitis. Graft usage compared to AVF access was associated with an increased risk of bacteremia, septicemia, conjunctivitis, and cellulitis. Alcohol dependence presented a heightened risk of bacteremia and septicemia despite a decreased risk of cellulitis. Black race compared to white race was associated with a decreased risk of bacteremia, septicemia, cellulitis, and herpes zoster. Hispanic ethnicity was associated with a decreased risk for all five infections.

## 4. Discussion

There is a lack of knowledge surrounding vitiligo and its role in increasing the risk of infection for patients with other comorbid conditions. In this study, we aimed to address this knowledge gap by evaluating the infection risk in patients with vitiligo undergoing dialysis for ESRD. Our analysis of the data revealed that vitiligo was diagnosed in 0.04% of the included patients with ESRD. Although the use of health insurance claims has previously been shown to demonstrate high diagnostic performance for vitiligo, it is worth noting that the lower proportion of patients identified within this population compared to national estimates may be attributable to underreporting among groups with higher prevalence (non-white) and/or due to the presentation of the disease (unilateral, segmental) [1,23].

The patients identified were more likely to be of black compared to white race, Hispanic ethnicity, female sex, a tobacco user, and/or with a hepatitis C diagnosis. Thus, our analysis demonstrated a significant association between vitiligo and race/ethnicity, with black and Hispanic persons being at a higher risk. While some studies have reported that vitiligo affects all ethnic groups equally, numerous studies have described potential reporting and diagnostic variances attributed to the greater visibility of vitiligo on darker skin tones [24,25]. These findings emphasize the importance of considering race and ethnicity in the diagnosis and treatment of vitiligo. Additionally, despite our finding of an elevated risk of vitiligo in females, previous epidemiological investigations have reported conflicting results regarding the gender predisposition. Some studies have identified no disparity between males and females, while others have reported conflicting findings regarding the prevalence of males versus females, with some studies indicating a higher prevalence in females and others suggesting a higher prevalence in males [26,27]. However, female patients may be more likely to seek early care due to the cosmetic and social implications of vitiligo, potentially contributing to the observed gender difference in our study [26]. Nevertheless, the prevalence of 0.04% observed in our study is low compared to the 0.76% to 1.11% prevalence of adult Americans determined in a recent study [1], although it should be noted that about 40% of these cases were reported to be undiagnosed. It is possible that the low prevalence is unique to the ESRD population or that underdiagnosis is even more of an issue in these patients compared to the general population. Alternatively, the use of ICD codes may result in undercounting, particularly as physicians treating these patients may be more concerned with the management of ESRD. However, our results, which show that vitiligo is an independent risk factor for certain infections, such as bacteremia and cellulitis, suggest the importance of physicians’ awareness of this diagnosis.

Tobacco use emerged as a significant clinical risk factor for vitiligo in our study. Proposed mechanisms for this observation relate to the production of reactive oxygen species (ROS) by the use of tobacco products [28,29]. However, it should be noted that other studies have suggested that tobacco use may suppress vitiligo, with tobacco smoke enhancing skin pigmentation in melanocytes [30,31], potentially by inhibiting the monoamine oxidases implicated in vitiligo pathogenesis [32]. Although the disparate results obtained in our study may relate to a particular population (those with ESRD), these contradictory results underscore the incomplete understanding of the effects of tobacco use in patients with vitiligo.

The hepatitis C virus (HCV) has been shown to trigger adult-onset vitiligo, potentially due to the deposition of immune complexes causing extrahepatic inflammatory responses, particularly in melanocytes and keratinocytes [33]. Thus, the association between HCV and the risk of vitiligo is perhaps not unexpected. Indeed, special consideration should be paid to HCV-infected patients receiving hemodialysis, since prior studies have shown increased rates of bacteremia and death compared to the general hemodialysis population [34].

Our analysis found an association between increasing age and a decreased risk of vitiligo. Studies have established that the incidence of vitiligo is highest among young individuals (10–30 years old) with nearly 80% developing the condition by age 30, and a decreasing incidence with increasing age, especially after the age of 50 [25,35]. One plausible explanation for this observation could be that the risk of developing autoimmune conditions like vitiligo decreases as individuals age. Vadasz et al. explains that the increase in protective immune mechanisms, such as an increased peripheral T-regulatory cell production found in the elderly, play a vital role in protection against development of autoimmune diseases [36].

Catheter access was correlated with a decreased likelihood of having vitiligo versus AVF access. Most of the literature to date recommends AVF as the first access type to be considered due to better outcomes with this type of access versus catheter access [37,38]. However, one study in 2013 documented disparities in AVF placement in older patients with hemodialysis, indicating that those of increasing age, female sex, and black race are less likely to receive AVF placement as their initial access type. In our study, all of these demographic factors were shown to be associated with an increased likelihood of a diagnosis of vitiligo [39], suggesting some confounding of this relationship.

In simple logistics regression models, vitiligo presented an increased risk of bacteremia, septicemia, cellulitis, and herpes zoster but not conjunctivitis. Controlling for demographics and clinical covariates determined that patients with ESRD with a vitiligo diagnosis had a significantly increased risk of a diagnosis of bacteremia, cellulitis, and herpes zoster but not septicemia or conjunctivitis. The increased risk for some infections may potentially be due to factors including disruption of the skin barrier, destruction of melanocytes, and dysregulation of autoimmune regulation in patients with vitiligo.

Vitiligo is characterized by the selective loss of melanocytes caused by autoimmune dysregulation, leading to the dyspigmentation of affected sites [25]. These areas of lesioned skin confer issues beyond cosmetic appearance, including delayed barrier recovery compared to uninvolved sites [14], which can impact barrier function and allow more ready entry of microorganisms. Indeed, keratinocytes in vitiligo lesions have been demonstrated to exhibit decreased levels of aquaporin 3, which is a water and glycerol channel that is known to be involved in epidermal barrier formation [40,41]. The levels of E-cadherin, which mediates keratinocyte–keratinocyte interactions, are also reduced [15]. The non-lesional skin of patients with vitiligo also shows altered epidermal lipid composition, with markedly decreased levels of ceramides, which are required for a competent permeability barrier [15]. In addition, vitiligo lesions also show impaired innate immunity. Thus, melanocytes can be mobilized by the activation of their innate immune pattern-recognition receptors in response to a host of extracellular bacteria and intracellular viral pathogens to promote the expression of type-I interferons (IFN α/β), cytokines (IL-1), and chemokines (CXCL-8/IL-8, CCL-2/MCP-1) [42]. Indeed, increased rates of hospitalization for herpes zoster have been observed among patients with chronic inflammatory skin conditions, including vitiligo [43].

Other parameters associated with one or more of the infections include increasing age, female sex, hemodialysis (versus peritoneal dialysis), catheter or graft versus AVF access, tobacco use, alcohol dependence, and hepatitis C infection. The association between increasing age and an increased risk of all five infections studied is not unexpected, as aged individuals show an increased susceptibility to infections [44,45]. Better outcomes with AVF use in patients undergoing dialysis are also expected based on previous studies [38]. Tobacco use was also associated with an increased risk of all the five infections studied. Tobacco induces physiological changes and immune system dysregulation [46,47,48]. Additionally, in patients undergoing dialysis, hepatitis C virus (HCV) infection and bacteremia are common comorbidities, and the presence of HCV further increased the risk of bacteremia, septicemia, and cellulitis, as was also indicated in our findings [49,50,51,52]. HCV weakens the immune system, allowing other pathogens introduced, e.g., via vascular access in dialysis patients, to thrive. Chronic alcohol use is also known to predispose people to the development of infections, especially bacteremia and sepsis [53]. Alcohol is known to downregulate the immune system and inhibit the release of the proinflammatory cytokines [53,54,55] needed to fight against pathogens. On the other hand, previous studies investigating gender differences in the risk of bacterial infections have yielded inconsistent results. Multiple studies have reported a higher incidence of bacterial infections, sepsis, and cellulitis in men compared to women [56,57,58,59], although the mechanisms are unclear.

While our study reports that black race and other races, compared to white race and Hispanic ethnicity are associated with a decreased risk of bacteremia, septicemia, and cellulitis, multiple previous studies have reported conflicting findings on this matter. One such study reported a higher incidence of bacterial infections and sepsis in black and Hispanic individuals even after controlling for income and geographic variances [60]. Similarly, another study reported a higher likelihood of cellulitis and other skin and soft tissue infections in African American and Hispanic individuals [61]. These differences may be due to underlying biological reasons, external factors, or the specific population examined (patients with ESRD).

Preventative measures most impactful for reducing infectious outcomes in this population may include obvious precautions, such as proper hand sanitization between patients, and education for healthcare professionals on the increased infection risk among individuals with vitiligo compared to patients with baseline ESRD, in conjunction with increased patient surveillance [62]. Other less apparent methods that could potentially reduce infection risk in ESRD patients with vitiligo include the use of flags in electronic medical records to highlight patients at risk for healthcare-associated infections. In addition, treatments to improve the epidermal barrier, as discussed in [63], may also prove beneficial.

This study has several limitations due to its reliance on the USRDS dataset. The diagnoses examined in this study were based on billing codes submitted to Medicare or derived from CMS Form 2728. It is important to note that these diagnoses were not based on actual clinical data. Consequently, it is difficult to determine the specialty or healthcare provider responsible for the billing or the extent of physical inspection by the physician. In addition, bias may be introduced as a result of the possibility that very light skin types may not consult a physician if experiencing depigmentation; as such, hypo- or depigmentation may not be as visible or troublesome, while those with darker skin types may be more motivated to seek help for pigmentation issues. Similarly, older patients may be less bothered or, due to flexibility issues, less aware of depigmented lesions and therefore may also be underdiagnosed. Further, it is important to note that this study lacks the ability to account for the clinical severity of vitiligo diagnosis, preventing the stratification of patients based on disease severity. Additionally, this study is unable to address potential coding idiosyncrasies, as well as instances of inaccurate or missed codes within the dataset. However, these limitations are somewhat mitigated by the large USRDS dataset, which captures billed diagnoses and therapies for all patients with ESRD in the United States, providing substantial statistical power for the analysis.

## 5. Conclusions

In conclusion, this study aimed to address the knowledge gap surrounding vitiligo and its association with an increased risk of infection for patients undergoing dialysis for ESRD. Those with a vitiligo diagnosis were more likely to be black, of Hispanic ethnicity, female, tobacco users, alcohol dependent, and/or with a hepatitis C diagnosis. Increasing age and catheter access were associated with a decreased risk of vitiligo. ESRD patients with vitiligo had an increased risk of bacteremia, cellulitis, and herpes zoster, potentially attributable to their disrupted skin barrier, melanocyte destruction, and immune system dysregulation. However, no significant association was found with septicemia or conjunctivitis. Overall, these findings highlight the importance of physician surveillance for infection in ESRD patients with vitiligo, although further research, preferably prospectively, is clearly warranted.

## Figures and Tables

**Table 1:** Descriptive statistics overall and by vitiligo, and logistic regression results on vitiligo.

Variable	Level	Overall	Vitiligo					
			Vitiligo	No Vitiligo	Simple Models		Final Model	
					RR (95%CI)	*p*-Value	aRR (95% CI)	*p*-Value
Age (years)—mean (SD)		63.5 (14.9)	60.6 (15.0)	63.5 (14.9)	0.99 (0.982–0.992)	<0.0001	0.992 (0.987–0.997)	0.0024
Race—n (%)	Black	425,522 (27.9)	245 (36.2)	425,277 (27.9)	1.45 (1.24–1.70)	<0.0001	1.51 (1.27–1.80)	<0.0001
	Other	93,724 (6.1)	32 (4.7)	93,692 (6.1)	0.86 (0.60–1.24)		1.07 (0.74–1.54)	
	White	1,007,024 (66.0)	399 (59.0)	1,006,625 (66.0)				
Sex—n (%)	Female	652,673 (42.8)	415 (61.4)	652,258 (42.8)	2.13 (1.8202.49)	<0.0001	2.41 (2.06–2.82)	<0.0001
	Male	873,597 (57.2)	261 (38.6)	873,336 (57.3)				
Ethnicity—n (%)	Hispanic	230,165 (15.1)	146 (21.6)	230,019 (15.1)	1.55 (1.29–1.86)	<0.0001	2.14 (1.75–2.62)	<0.0001
	Non-Hispanic	1,296,105 (84.9)	530 (78.4)	1,295,575 (84.9)				
Dialysis Modality—n (%)	HD	1,525,481 (99.9)	676 (100.0)	1,524,805 (99.9)				
	PD	789 (0.1)	0 (0.0)	789 (0.1)				
Access Type—n (%)	Catheter	1,232,849 (80.8)	503 (74.4)	1,232,346 (80.8)	0.71 (0.59–0.85)	0.0001	0.65 (0.54–0.78)	<0.0001
	Graft	49,771 (3.3)	32 (4.7)	49,739 (3.3)	1.11 (0.76–1.63)		0.90 (0.61–1.33)	
	AVF	243,650 (16)	141 (20.9)	243,509 (16)				
Tobacco—n (%)	Yes	273,815 (17.9)	286 (42.3)	273,529 (17.9)	3.36 (2.88–3.91)	<0.0001	3.54 (3.02–4.15)	<0.0001
	No	1,252,455 (82.1)	390 (57.7)	1,252,065 (82.1)				
Alcohol Dependence—n (%)	Yes	42,412 (2.8)	31 (4.6)	42,381 (2.8)	1.68 (1.17–2.41)	0.0047		
	No	1,483,858 (97.2)	645 (95.4)	1,483,213 (97.2)				
Hepatitis C—n (%)	Yes	33,859 (2.2)	49 (7.3)	33,810 (2.2)	3.45 (2.58–4.61)	<0.0001	2.14 (1.59–2.89)	<0.0001
	No	1,492,411 (97.8)	627 (92.8)	1,491,784 (97.8)				

Note: Black-shaded cells indicate that a variable was not examined due to zero frequencies in simple models or that a variable did not remain in the final model. RR = relative risk, aRR = adjusted relative risk, CI = confidence interval, HD = hemodialysis, PD = peritoneal dialysis, AVF = arteriovenous fistula.

**Table 2:** Descriptive statistics by infectious outcomes.

Variable	Level	Bacteremia		Septicemia		Cellulitis		Herpes Zoster		Conjunctivitis	
		Yes	No	Yes	No	Yes	No	Yes	No	Yes	No
Main Independent Variable											
Vitiligo—n (%)	Yes	227 (0.1)	449 (0.0)	475 (0.1)	201 (0.0)	331 (0.1)	345 (0.0)	50 (0.1)	626 (0.0)	14 (0.1)	662 (0.0)
	No	236,354 (99.9)	1,289,240 (100.0)	548,890 (99.9)	976,704 (100.0)	362,695 (99.9)	1,162,899 (100.0)	39,842 (99.9)	1,485,752 (100.0)	15,391 (99.9)	1,510,203 (100.0)
Demographic and Clinical Risk Factors											
Age (years) —mean (SD)		62.3 (14.8)	63.7 (14.8)	64.5 (14.3)	63 (15.1)	62.5 (14.3)	63.8 (15)	63.6 (14.5)	63.5 (14.9)	61.2 (16.1)	63.5 (14.8)
Race—n (%)	Black	78,047 (33.0)	347,475 (26.9)	159,633 (29.1)	265,889 (27.2)	91,859 (25.3)	333,663 (28.7)	9531 (23.9)	415,991 (28)	5032 (32.7)	420,490 (27.8)
	Other	11,648 (4.9)	82,076 (6.4)	30,738 (5.6)	62,986 (6.5)	17,575 (4.8)	76,149 (6.6)	2661 (6.7)	91,063 (6.1)	979 (6.4)	92,745 (6.1)
	White	146,886 (62.1)	860,138 (66.7)	358,994 (65.4)	648,030 (66.3)	253,592 (69.9)	753,432 (64.8)	27,700 (69.4)	979,324 (65.9)	9394 (61)	997,630 (66.0)
Sex—n (%)	Female	106,191 (44.9)	546,482 (42.4)	247,471 (45.1)	405,202 (41.5)	160,396 (44.2)	492,277 (42.3)	20,027 (50.2)	632,646 (42.6)	7676 (49.8)	644,997 (42.7)
	Male	130,390 (55.1)	743,207 (57.6)	301,894 (55)	571,703 (58.5)	202,630 (55.8)	670,967 (57.7)	19,865 (49.8)	853,732 (57.4)	7729 (50.2)	865,868 (57.3)
Ethnicity—n (%)	Hispanic	29,515 (12.5)	200,650 (15.6)	73,920 (13.5)	156,245 (16)	51,711 (14.2)	178,454 (15.3)	5589 (14.0)	224,576 (15.1)	2219 (14.4)	227,946 (15.1)
	Non-Hispanic	207,066 (87.5)	1,089,039 (84.4)	475,445 (86.5)	820,660 (84)	311,315 (85.8)	984,790 (84.7)	34,303 (86.0)	1,261,802 (84.9)	13,186 (85.6)	1,282,919 (84.9)
Dialysis Modality—n (%)	HD	236,482 (100.0)	1,288,999 (100.0)	549,115 (100.0)	976,366 (99.9)	362,870 (100.0)	1,162,611 (100.0)	39,867 (99.9)	1,485,614 (100.0)	15,399 (100.0)	1,510,082 (100.0)
	PD	99 (0.0)	690 (0.1)	250 (0.1)	539 (0.1)	156 (0.0)	633 (0.1)	25 (0.1)	764 (0.1)	NR *	NR *
Access Type—n (%)	Catheter	199,842 (84.5)	1,033,007 (80.1)	458,253 (83.4)	774,596 (79.3)	294,500 (81.1)	938,349 (80.7)	31,285 (78.4)	1,201,564 (80.8)	12,488 (81.1)	1,220,361 (80.8)
	Graft	8482 (3.6)	41,289 (3.2)	18,313 (3.3)	31,458 (3.2)	12,724 (3.5)	37,047 (3.2)	1409 (3.5)	48,362 (3.3)	621 (4)	49,150 (3.3)
	AVF	28,257 (11.9)	215,393 (16.7)	72,799 (13.3)	170,851 (17.5)	55,802 (15.4)	187,848 (16.2)	7198 (18)	236,452 (15.9)	2296 (14.9)	241,354 (16.0)
Tobacco —n (%)	Yes	64,343 (27.2)	209,472 (16.2)	162,088 (29.5)	111,727 (11.4)	102,902 (28.4)	170,913 (14.7)	11,264 (28.2)	262,551 (17.7)	3643 (23.7)	270,172 (17.9)
	No	172,238 (72.8)	1,080,217 (83.8)	387,277 (70.5)	865,178 (88.6)	260,124 (71.7)	992,331 (85.3)	28,628 (71.8)	1,223,827 (82.3)	11,762 (76.4)	1,240,693 (82.1)
Alcohol Dependence—n (%)	Yes	8524 (3.6)	33,888 (2.6)	20,397 (3.7)	22,015 (2.3)	11,971 (3.3)	30,441 (2.6)	1192 (3.0)	41,220 (2.8)	470 (3.0)	41,942 (2.8)
	No	228,057 (96.4)	1,255,801 (97.4)	528,968 (96.3)	954,890 (97.8)	351,055 (96.7)	1,132,803 (97.4)	38,700 (97.0)	1,445,158 (97.2)	14,935 (97.0)	1,468,923 (97.2)
Hepatitis C—n (%)	Yes	11,770 (5.0)	22,089 (1.7)	25,695 (4.7)	8164 (0.8)	15,654 (4.3)	18,205 (1.6)	1571 (3.9)	32,288 (2.2)	596 (3.9)	33,263 (2.2)
	No	224,811 (95.0)	1,267,600 (98.3)	523,670 (95.3)	968,741 (99.2)	347,372 (95.7)	1,145,039 (98.4)	38,321 (96.1)	1,454,090 (97.8)	14,809 (96.1)	1,477,602 (97.8)

* NR = Not reported because—per USRDS regulations—values of <11 must be suppressed. HD = hemodialysis, PD = peritoneal dialysis, AVF = arteriovenous fistula.

**Table 3:** Logistic regression results of vitiligo on infectious outcomes.

Variable	Level	Simple Models: RR (95% CI) *p*-Value					Final Models: aRR (95% CI) *p*-Value				
		Bacteremia	Septicemia	Cellulitis	Herpes Zoster	Conjunctivitis	Bacteremia	Septicemia	Cellulitis	Herpes Zoster	Conjunctivitis
Main Independent Variable											
Vitiligo	Yes vs. No	1.28 (1.13–1.46) 0.0002	1.20 (1.10–1.31) <0.0001	1.27 (1.15–1.42) <0.0001	1.69 (1.28–2.22) 0.0002	1.22 (0.72–2.06) 0.4608	1.20 (1.05–1.37) 0.0063	1.08 (0.98–1.18) 0.1169	1.15 (1.03–1.28) 0.0129	1.51 (1.14–1.99) 0.0040	1.13 (0.67–1.90) 0.6593
Demographic and Clinical Risk Factors											
Age (years)	1 year increase	1.016 (1.015–1.016) <0.0001	1.027 (1.027–1.028) <0.0001	1.017 (1.017–1.017) <0.0001	1.023 (1.022–1.024) <0.0001	1.011 (1.010–1.013) <0.0001	1.016 (1.016–1.016) <0.0001	1.028 (1.027–1.028) <0.0001	1.015 (1.014–1.015) <0.0001	1.021 (1.020–1.022) <0.0001	1.011 (1.010–1.012) <0.0001
Race	Black vs. White	1.03 (1.02–1.04) <0.0001	0.85 (0.85–0.86) <0.0001	0.66 (0.66–0.67) <0.0001	0.64 (0.62–0.65) <0.0001	1.00 (0.97–1.04) 0.8865	0.98 (0.97–0.99) <0.0001	0.87 (0.87–0.88) <0.0001	0.63 (0.63–0.64) <0.0001	0.64 (0.62–0.65) <0.0001	0.97 (0.94–1.01) 0.1050
	Other vs. White	0.68 (0.66–0.69) <0.0001	0.73 (0.72–0.74) <0.0001	0.57 (0.56–0.58) <0.0001	0.83 (0.80–0.87) <0.0001	0.91 (0.85–0.97) 0.0038	0.67 (0.65–0.68) <0.0001	0.77 (0.76–0.78) <0.0001	0.57 (0.56–0.57) <0.0001	0.84 (0.81–0.87) <0.0001	0.89 (0.83–0.95) 0.0007
Sex	Female vs. Male	1.12 (1.11–1.13) <0.0001	1.14 (1.13–1.15) <0.0001	1.09 (1.08–1.10) <0.0001	1.38 (1.35–1.40) <0.0001	1.35 (1.31–1.39) <0.0001	1.10 (1.09–1.11) <0.0001	1.12 (1.12–1.13) <0.0001	1.11 (1.10–1.12) <0.0001	1.40 (1.37–1.43) <0.0001	1.33 (1.29–1.37) <0.0001
Ethnicity	Hispanic vs. Non-Hispanic	0.66 (0.65–0.66) <0.0001	0.71 (0.71–0.72) <0.0001	0.77 (0.76–0.78) <0.0001	0.76 (0.74–0.78) <0.0001	0.79 (0.75–0.83) <0.0001	0.68 (0.67–0.69) <0.0001	0.77 (0.77–0.78) <0.0001	0.71 (0.70–0.72) <0.0001	0.74 (0.72–0.77) <0.0001	0.82 (0.78–0.86) <0.0001
Dialysis Modality	HD vs. PD	1.37 (1.13–1.67) 0.0016	1.28 (1.13–1.45) 0.0001	1.39 (1.19–1.63) <0.0001	0.89 (0.61–1.31) 0.5724	1.44 (1.65–3.21) 0.3738	1.28 (1.05–1.56) 0.0157	1.14 (1.01–1.30) 0.0323	1.37 (1.17–1.6) <0.0001		
Access Type	Catheter vs. AVF	1.85 (1.82–1.87) <0.0001	1.70 (1.68–1.71) <0.0001	1.35 (1.33–1.36) <0.0001	1.08 (1.05–1.11) <0.0001	1.35 (1.29–1.41) <0.0001	1.91 (1.89–1.94) <0.0001	1.80 (1.78–1.81) <0.0001	1.42 (1.41–1.43) <0.0001	1.12 (1.09–1.15) <0.0001	1.36 (1.30–1.42) <0.0001
	Graft vs. AVF	1.69 (1.64–1.73) <0.0001	1.43 (1.41–1.46) <0.0001	1.27 (1.25–1.30) <0.0001	1.05 (0.99–1.11) 0.0814	1.46 (1.33–1.59) <0.0001	1.59 (1.55–1.63) <0.0001	1.34 (1.32–1.37) <0.0001	1.29 (1.26–1.32) <0.0001	1.01 (0.95–1.07) 0.7636	1.34 (1.23–1.47) <0.0001
Tobacco	Yes vs. No	1.46 (1.45–1.48) <0.0001	1.73 (1.72–1.74) <0.0001	1.60 (1.59–1.61) <0.0001	1.51 (1.48–1.55) <0.0001	1.19 (1.14–1.23) <0.0001	1.38 (1.37–1.39) <0.0001	1.67 (1.66–1.68) <0.0001	1.54 (1.53–1.55) <0.0001	1.54 (1.51–1.57) <0.0001	1.19 (1.14–1.23) <0.0001
Alcohol Dependence	Yes vs. No	1.22 (1.19–1.24) <0.0001	1.28 (1.26–1.29) <0.0001	1.11 (1.09–1.13) <0.0001	0.99 (0.93–1.05) 0.6597	1.01 (0.92–1.11) 0.8528	1.04 (1.01–1.05) 0.0028	1.10 (1.08–1.11) <0.0001	0.95 (0.93–0.97) <0.0001		
Hepatitis C	Yes vs. No	1.90 (1.87–1.94) <0.0001	1.91 (1.88–1.94) <0.0001	1.67 (1.64–1.70) <0.0001	1.39 (1.32–1.46) <0.0001	1.36 (1.25–1.47) <0.0001	1.74 (1.70–1.77) <0.0001	1.73 (1.70–1.75) <0.0001	1.61 (1.58–1.63) <0.0001	1.43 (1.36–1.51) <0.0001	1.35 (1.24–1.47) <0.0001

Note: Black-shaded cells indicate that a variable did not remain in the final model. RR = relative risk, aRR = adjusted relative risk, CI = confidence interval, HD = hemodialysis, PD = peritoneal dialysis, AVF = arteriovenous fistula.

## Data Availability

The data underlying this article were provided by the United States Renal Data System (USRDS) under a data use agreement. Data will be shared upon request with permission of the United States Renal Data System.
